# Pragmatic physiologically-based pharmacokinetic modeling to support clinical implementation of optimized gentamicin dosing in term neonates and infants: proof-of-concept

**DOI:** 10.3389/fped.2023.1288376

**Published:** 2023-11-21

**Authors:** Marika A. de Hoop-Sommen, Joyce E. M. van der Heijden, Jolien J. M. Freriksen, Rick Greupink, Saskia N. de Wildt

**Affiliations:** ^1^ Division of Pharmacology and Toxicology, Department of Pharmacy, Radboud University Medical Center, Nijmegen, Netherlands; ^2^ Department for Intensive Care, Radboud University Medical Center, Nijmegen, Netherlands; ^3^ Intensive Care and Pediatric Surgery, Erasmus MC, Rotterdam, Netherlands

**Keywords:** term neonate, infant, PBPK, model-informed dose, gentamicin, pediatric pharmacology, clinical implementation

## Abstract

**Introduction:**

Modeling and simulation can support dosing recommendations for clinical practice, but a simple framework is missing. In this proof-of-concept study, we aimed to develop neonatal and infant gentamicin dosing guidelines, supported by a pragmatic physiologically-based pharmacokinetic (PBPK) modeling approach and a decision framework for implementation.

**Methods:**

An already existing PBPK model was verified with data of 87 adults, 485 children and 912 neonates, based on visual predictive checks and predicted-to-observed pharmacokinetic (PK) parameter ratios. After acceptance of the model, dosages now recommended by the Dutch Pediatric Formulary (DPF) were simulated, along with several alternative dosing scenarios, aiming for recommended peak (i.e., 8–12 mg/L for neonates and 15–20 mg/L for infants) and trough (i.e., <1 mg/L) levels. We then used a decision framework to weigh benefits and risks for implementation.

**Results:**

The PBPK model adequately described gentamicin PK. Simulations of current DPF dosages showed that the dosing interval for term neonates up to 6 weeks of age should be extended to 36–48 h to reach trough levels <1 mg/L. For infants, a 7.5 mg/kg/24 h dose will reach adequate peak levels. The benefits of these dose adaptations outweigh remaining uncertainties which can be minimized by routine drug monitoring.

**Conclusion:**

We used a PBPK model to show that current DPF dosages for gentamicin in term neonates and infants needed to be optimized. In the context of potential uncertainties, the risk-benefit analysis proved positive; the model-informed dose is ready for clinical implementation.

## 1. Introduction

Gentamicin is a widely used aminoglycoside antibiotic used for infections with gram-negative and gram-positive bacteria, such as in pneumonia, urinary tract infections, and sepsis. It is a hydrophilic drug that is excreted renally with a half-life of 2–3 h in adults. Initially, gentamicin was registered for multiple daily dosing, although its post antibiotic effect makes it suitable for once-daily dosing. Furthermore, a reduced risk of bacterial resistance and diminished accumulation in the renal tubules and inner ear, both target organs for toxicity, have been observed after once-daily dosing (1–4). Despite the current consensus on once-daily dosing, drug labels still differ in this regard (5, 6). The U.S. label still recommends the traditional thrice-daily dosing, while the European label prefers once-daily over twice-daily dosing (7–9). Also, there is no consensus with regard to the therapeutic targets, especially in special populations like pediatric, obese, and elderly patients (5, 10, 11).

Next to differences in dosing frequency and therapeutic targets, the differences in recommended dosages are also substantial among dosing guidelines and drug labels. This has been indicated specifically for neonates, but these differences also exist for infants, children and adolescents (Supplementary Table S1) (12, 13). In addition, the evidence used for the current doses in the Dutch Pediatric Formulary (DPF) in neonates and infants is limited; only 4 references were cited, 2 of which are very specific to children with febrile neutropenia, aiming at peak concentrations (*C*_max_) of at least 20 or 25 mg/L (14, 15). Another study aiming at similarly high *C*_max_ values, proposed a dose of 9.5 mg/kg every 24 h (16). The fourth study, a population pharmacokinetic (popPK) study, suggested a dose of 7 mg/kg for infants to reach a *C*_max_ of at least 10 mg/L, which is the currently recommended DPF dose (17). However, all 4 of these trials aimed at a different therapeutic window than suggested by therapeutic drug monitoring (TDM) guidelines, so the evidence base for this age category needed strengthening.

Many studies have been conducted to find “the” right dose (18, 19). Especially in preterm neonates, several popPK models have been developed, and also term neonates in their first week of life were extensively studied (19). Older neonates and young infants, however, are less studied, despite reported large dose ranges of 4.5–7.5 mg/kg every 24 h (19, 20). This variation in dosing partially reflects the wide variability in pharmacokinetics (PK), as body composition and organ maturation change tremendously during neonatal and infant periods (21). For example, gentamicin's volume of distribution (Vd) decreases from 0.48 L/kg in a preterm neonate to 0.35 L/kg in an infant, while clearance (CL) nearly triples from neonatal to infant age (19). Yet, this is a gradual process, where the dose certainly needs to be adjusted over time, but the question remains of exactly how and when to adjust it. Dose increases of 33%–52% (Supplementary Table S1) are partly explained by the change in therapeutic target (from 8–12 mg/L to 15–20 mg/L), but it is questionable if such a large, abrupt dose increase is indeed justified.

Pediatric physiologically-based pharmacokinetic (PBPK) models capture the developmental changes in PK related processes. Such models have been previously developed for gentamicin, but they did not aim to provide dosing guidelines or infants were not represented in these models (Supplementary Table S2) (22–25). At the same time abundant gentamicin PK data from neonates (i.e., 0–28 days postnatal age) and infants (i.e., 1–24 months of age) are widely available, enabling verification of dosing simulations. Previously, we have shown that PBPK modeling is feasible for the prediction of pediatric exposure of seven drugs and have also published a tutorial outlining a pragmatic approach to this methodology (26, 27). We now aim to expand this to establish pediatric doses for clinical implementation. Hence, the aim of our study was to pragmatically establish rational gentamicin doses for term neonates and young infants, while also assessing the risks and benefits of these model-informed doses for clinical implementation.

## 2. Methods

The PBPK platform Simcyp™ Simulator (version 21; Certara, Sheffield, UK) was used for our simulations. For our pragmatic approach, we compared the two recently published gentamicin PBPK models developed in Simcyp™ using exploratory simulations (23, 24). Gentamicin *C*_max_ and trough concentration (*C*_trough_) were used as surrogate markers for efficacy and toxicity. The model that best predicted gentamicin PK, and more specifically *C*_max_ and *C*_trough_, was the full distribution model reported by Abduljalil et al. (23). Next, the performance of this model was assessed according to the approach described by van der Heijden et al., using published adult and pediatric PK data (26).

### 2.1. Model verification

To verify the model, we searched for published PK data that were not used in model development by Abduljalil et al. (Supplementary Tables S3a–c). In simulations, default Simcyp™ populations were used, for adults we used the “Sim-healthy volunteer” population or “Sim-NEurCaucasian”, in case patients above 65 years of age were included (28, 29). For children, both the “Sim-paediatric” and the “Sim-preterm” populations were used (23, 30, 31). Trial design (e.g., administered dose, age range, proportion of females, and trial duration) was matched to the corresponding clinical study, using ten trials of ten virtual subjects. For children younger than 1 year, the “redefining subjects over time” option was activated, to allow for virtual growth (physiologically and biochemically) of the virtual subjects during the simulation. Model performance was assessed by visual predictive checks (VPC) and by calculating predicted-to-observed (P/O) PK parameter ratios. Ratios within 2-fold were considered acceptable, the closer the ratio was to 1, the better the prediction. The ratios of surrogate markers *C*_max_ and *C*_trough_ were also compared to the bio-equivalence range (i.e., 1.25-fold).

### 2.2. Dose simulations

Prior to the dose simulations, we had to define the therapeutic targets. For efficacy, the most commonly used therapeutic targets are the *C*_max_ or the area under the curve (AUC), where the ratio to the minimal inhibitory concentration (MIC) of a bacterium should be within a certain range. For our target population, *C*_max_/MIC is the best therapeutic target, as a study in neonates found that AUC/MIC was not a determining factor for the efficacy of gentamicin (32). Moreover, most guidelines for gentamicin use *C*_max_ and agree that a *C*_max_/MIC ratio of 8–10 should be aimed for (33–36). In neonatal infections, multiple studies have shown that most micro-organisms involved in these infections have an MIC of less than 1 mg/L (33, 36–40), which has been translated into a therapeutic window of 8–12 mg/L for *C*_max_ in most guidelines. For all other age groups, i.e., infants through adults, a *C*_max_ between 15 and 20 mg/L is recommended, as the infections treated with gentamicin are often caused by micro-organisms with MICs up to 2 mg/L (33, 35, 36, 41).

For toxicity, both AUC and *C*_trough_ have been used (42–45). However, the upper threshold for daily or cumulative AUC for the occurrence of nephrotoxicity is currently based on multiple daily dosing (45), and no AUC based thresholds are known for once-daily dosing. Therefore, we assumed that *C*_trough_ is a better predictor of toxicity in this study. A range of maximum trough levels of 0.5–2 mg/L has been used as target threshold (33–36, 46), though qualitative research is currently lacking whether a *C*_trough_ of 2 mg/L results in more toxicity than 1 or 0.5 mg/L (42). Also drug labels differ in this regard; the U.S. label recommends a *C*_trough_ ≤2 mg/L, while the EU label differentiates between a trough level for twice-daily administration (≤2 mg/L) and once-daily administration (≤1 mg/L) (7–9). As such, we used a *C*_trough_ of 1 mg/L as a predictor of toxicity in this study.

To find the best model-informed dose, we simulated several dosing scenarios. For our age group of interest (term neonates—infants), these simulations were performed using the “Sim-Paediatric” population, using ten trials of ten virtual subjects. The current DPF dosing recommendations for term neonates (4 mg/kg every 24 h) and infants (7 mg/kg every 24 h) were simulated first, followed by simulations of several alternative dosing scenarios (Supplementary Table S4). Gentamicin was simulated as a single, intravenous infusion over 30 min for a 48-h period, as dosing thereafter is usually based on therapeutic drug monitoring (TDM) if longer therapy is needed. For each simulation, we assessed *C*_max_, *C*_trough_ and the corresponding 5th and 95th percentiles (33, 34). In accordance with various TDM guidelines, also in our simulations *C*_max_ was taken at 1 h after the start of infusion (i.e., 30 min after the end of the 30 min infusion period) and *C*_trough_ 30 min before the next (theoretical) dose (33, 36). For neonates, we assessed *C*_trough_ at 23.5, 35.5 and 47.5 h after the start of infusion in order to extensively evaluate trough levels, and assess the potential need for an extended dosing interval. For infants, *C*_trough_ was assessed only at 23.5 h after the start of infusion as no elevated trough levels were expected based on literature. When the 5th to 95th percentile for simulated *C*_max_ and/or *C*_trough_ fell outside the therapeutic window for its specific ages [i.e., *C*_max_ within 8–12 mg/L for neonates and within 15–20 mg/L for infants, *C*_trough_ below 1 mg/L for both neonates and infants (33)], the dose and/or dose frequency was further adjusted in the simulations until desired levels were reached (Supplementary Table S4).

### 2.3. Clinical implementation

After completing model verification and conducting dose simulations of current DPF doses and alternative dosing regimens, we interpreted our simulation results in context of a previously published framework for implementing model-informed doses in a clinical setting (47). The following questions were addressed:

1. What is the level of certainty on the target concentrations?
2. What is the clinical risk of over- or underdosing?
3. What is the level of certainty of the model output?
4. Does the currently advised DPF dose result in adequate target exposure?
5. Which dose results in better target exposure, is this a significant improvement?
6. Is the proposed dose practical?
7. Is the population used to verify the PBPK model comparable to the intended population (e.g., with respect to demographics, severity of illness, underlying disease)? If not, will this impact the dosing requirements?
8. What is the overall conclusion for gentamicin?

Based on these questions, we derived model-informed dosing recommendations along with a recommendation for implementation in clinical practice.

## 3. Results

### 3.1. Model verification

**Figure 1:**
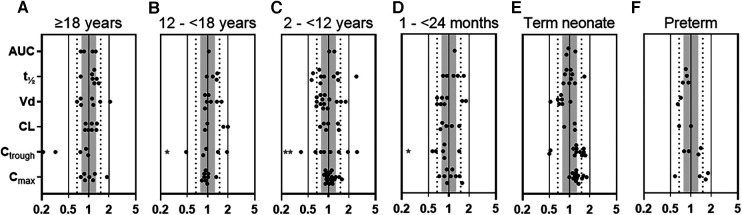
Predicted-to-observed PK parameter ratios for gentamicin, separated for each age group (i.e., (**A**) adults ≥18 years, (**B**) adolescents 12–<18 years, (**C**) children 2–<12 years, (**D**) infants 1–<24 months, (**E**) term neonates 0–28 days postnatal age, and (**F**) preterm neonates 0–28 days postnatal age). Solid lines, dotted lines, and shaded area indicate the 2-fold, 1.5-fold, and 1.25-fold range, respectively. * 1 or 2 (**) datapoints fell outside axis limits. AUC, area under the curve; t_½_, half-life; Vd, volume of distribution; CL, clearance, *C*_trough_, plasma trough concentration; *C*_max_, maximal plasma concentration.

To verify the gentamicin PBPK model in adults, pediatrics, and preterms, we used published PK data of 8 adult, 19 pediatric, and 3 preterm studies, representing 87, 485, and 912 patients, respectively (Supplementary Tables S3a–c). For adults, 82% of all calculated P/O PK parameter ratios were within 2-fold and for pediatric patients this percentage was as high as 91%. More specifically, for pediatric *C*_max_ and *C*_trough_ values, the P/O ratios were within 2-fold for 100% (*n *=* *54) and 82% (*n *=* *39), and within 1.25-fold for 65% and 29%, respectively (Figure 1 and Supplementary Table S5). Although in term neonates a slight overprediction of *C*_max_ and *C*_trough_ was visible, this fell within the prespecified acceptance ranges, as illustrated in Figures 1 and Supplementary Figures S1–S3.

### 3.2. Dose simulations

**Figure 2:**
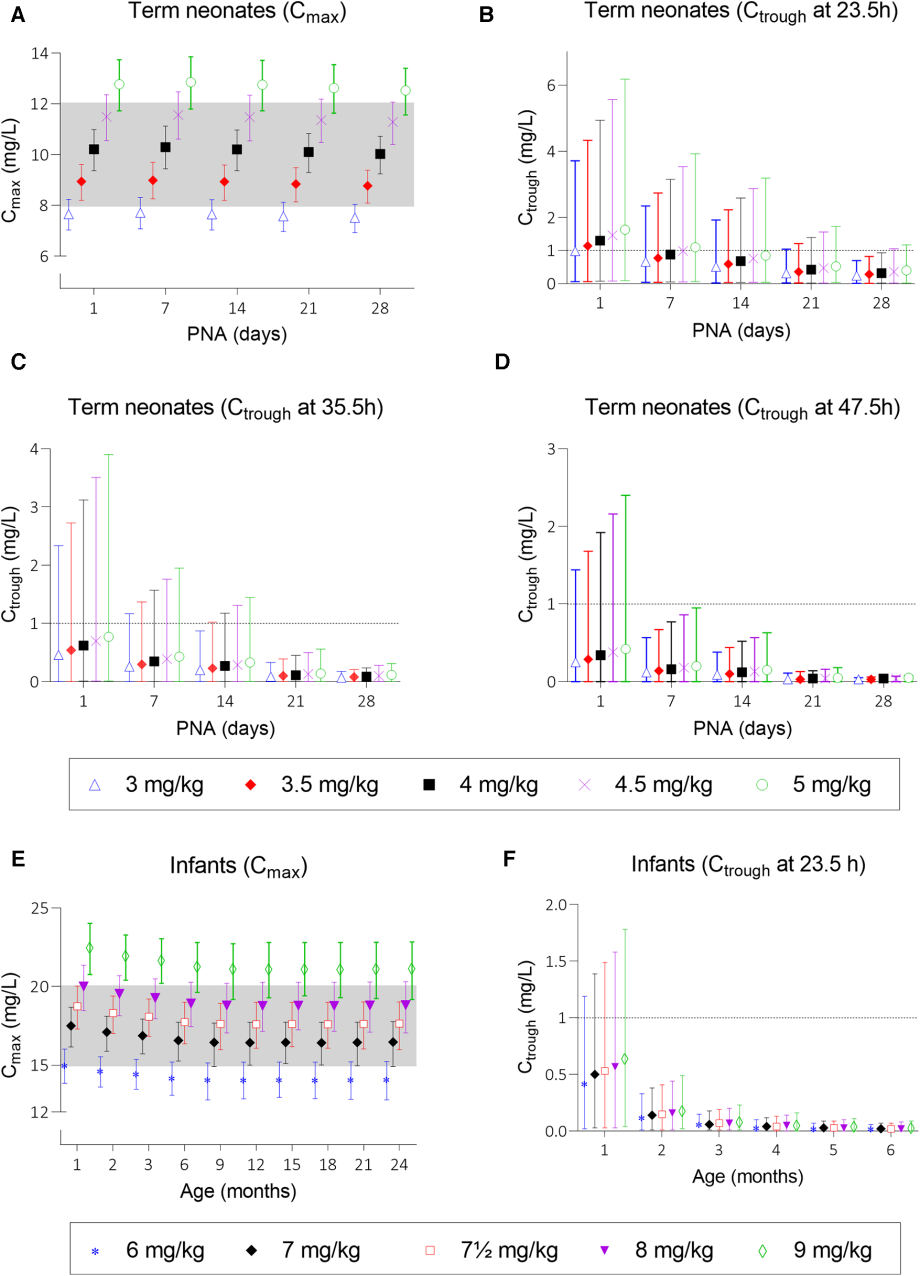
Mean predicted *C*_max_ and *C*_trough_ levels and their 5th and 95th percentiles at several dosing scenarios. Graph (**A**) shows *C*_max_ levels after doses of 3, 3.5, 4, 4.5, and 5 mg/kg for neonates and graph (**E**) shows *C*_max_ levels after doses of 6, 7, 7.5, 8, and 9 mg/kg for infants. Graph (**B**–**D**) show *C*_trough_ levels at 23.5, 35.5, and 47.5 h after start of infusion for neonates and graph (**F**) shows *C*_trough_ levels for infants until the age of 6 months; older infants all have trough levels below 1 mg/L and are therefore not depicted. Black squares (▪) in graphs (**A**–**D**) and black diamonds (◆) in graphs (**E,F**) represent the Dutch Pediatric Formulary dose. PNA, postnatal age.

**Figure 3:**
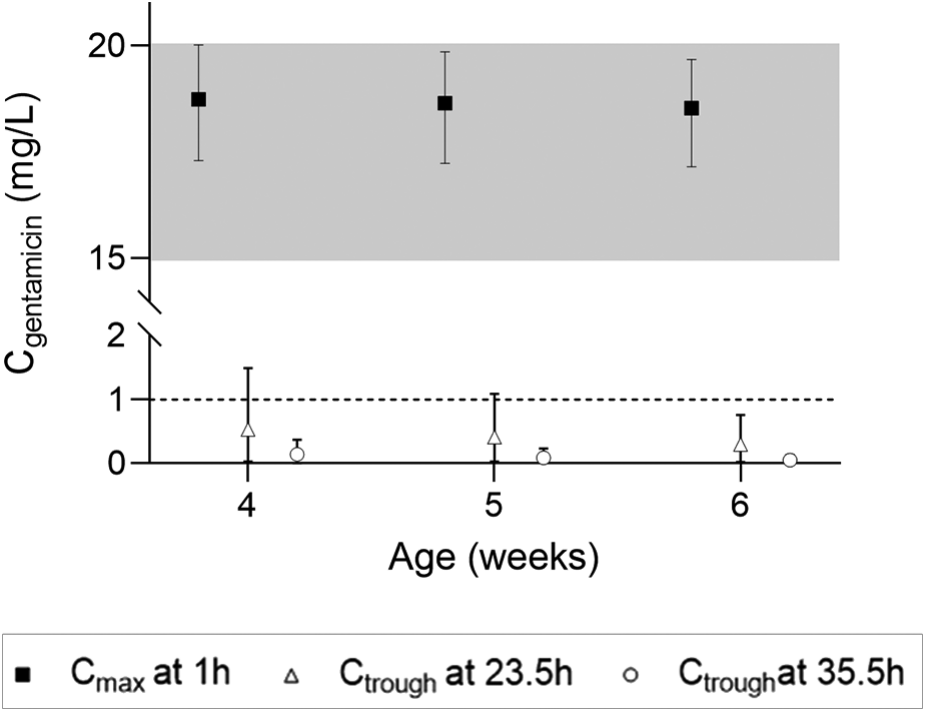
*C*_max_ and *C*_trough_ levels and their 5th and 95th percentiles in infants 4–6 weeks of age upon a 7.5 mg/kg dose.

Simulations of the current DPF dosing recommendations for term neonates showed that maximal concentrations fell within the therapeutic window with a median *C*_max_ of 10.2 mg/L (Figure 2A, i.e., black squares). On the contrary, *C*_trough_ only fell below 1 mg/L for neonates approximating 1 month of age (Figure 2B, i.e., black squares). For infants, almost all simulated *C*_max_ levels were within the therapeutic window, although the 5th percentile for infants ≥21 months of age just dipped below the lower limit (Figure 2E, i.e., black diamonds). Similarly, the DPF dose led to adequate trough levels, except in one-month-old infants. In these children, the 95th percentile was above 1 mg/L (Figure 2F, i.e., black diamonds). Further simulations showed that from 6 weeks of age onwards, this 95th percentile would be below 1 mg/L again. Our simulations indicated that for infants 4–6 weeks of age an extended interval of 36 h was required to fulfil the target requirements (Figure 3).

Taking these findings as a starting point, we simulated a series of potential alternatives to the current DPF dosing scenarios in order to optimize gentamicin exposure (Supplementary Table S4). As for most neonates *C*_trough_ levels at 23.5 h after dosing were too high, the dose interval was extended (36–48 h). Although neonatal *C*_max_ was predicted in the middle of the therapeutic window, we simulated several other dosages to assess the effect on *C*_trough_. Figures 2A–D show the mean predicted *C*_max_ and *C*_trough_ levels with the 5th to 95th percentiles for each dosing regimen.

Based on these simulations, initial model-informed dosing recommendations could be derived. For example, the majority of the one-day-old neonates did not reach a *C*_trough_ below 1 mg/L when a dose is administered every 24 h (Figure 2B). When administered every 48 h, at least 95% of the predicted population is expected to reach trough levels below 1 mg/L. Regarding *C*_max_, dose simulations showed that for a one-day-old child, doses of 3.5 and 4 mg/kg every 24 h resulted in adequate concentrations, as both the mean *C*_max_ and the 5th and 95th percentiles were within the therapeutic range. In case several simulated doses seemed adequate, the highest dose was chosen for the sake of efficacy. For each simulated age, we made similar trade-offs (Supplementary Table S6) with the final model-informed dosing recommendations shown in Table 1.

**Table 1:** Final model-informed dosing recommendations for neonates and infants.

Age	Final dose recommendation
<21 days	4 mg/kg every 48 h
21–28 days	4 mg/kg every 36 h
28–42 days	7.5 mg/kg every 36 h
Infants 6 weeks–2 years	7.5 mg/kg every 24 h

### 3.3. Clinical implementation

Before proceeding to clinical application of these model-informed doses, we weighted uncertainties, by placing our simulation results in the context of the framework proposed by Hartman et al. (47). The following questions were addressed:

1. *What is the level of certainty on the target concentrations?* We assessed this as “high certainty”, based on a widely accepted *C*_max_/MIC ratio of 8–10 for infections with bacteria with an MIC of ≤1 mg/L for neonates and ≤2 mg/L for infants (33–36).
2. *What is the clinical risk of over- or underdosing?* Gentamicin's main concerns are its oto- and nephrotoxicity, which can be minimized by keeping through concentrations <1 mg/L. Based on our simulations we evaluated the risk as low. When only 1 dose is administered, this is not of concern, but when gentamicin is continued, measuring trough levels is part of routine TDM, at least in most clinical guidelines, and reduces the risk of dose-related toxicity due to increased trough levels. While underdosing may result in ineffective treatment, potentially worsening the disease state with an increased risk of death, our simulated dosages support a low risk of underdosing. Also, the risk of underdosing is reduced by routine TDM in case of continued use.
3. *What is the level of certainty of the model output?* High, as model performance could be verified against several available clinical data sets and model performance was robust, resulting in a high certainty of model outcomes.
4. *Does the currently advised DPF dose result in adequate target exposure?* No, simulations showed that the current neonatal DPF dose resulted in excessive trough levels, while the infant dose does not always result in adequate peak levels. This was supported by Hartman et al., who showed that 30% of term neonates had excessive trough levels and 87% of infants did not reach therapeutic levels (48).
5. *Which dose results in better target exposure, is this a significant improvement?* The extended dosing interval results in better trough levels for neonates. The model-supported increase in dose for infants prevents underdosing of *C*_max_ when infected by a micro-organism with an MIC of ≤2 mg/L.
6. *Is the proposed dose practical?* Yes, it is acceptable, although there are now 4 instead of 2 dosing recommendations for neonates and infants.
7. *Is the population used to verify the PBPK model comparable to the intended population (e.g., with respect to demographics, severity of illness, underlying disease)? If not, will this impact the dosing requirements?* Yes, for model verification, we mostly used PK studies with patients of all ages, treated for various diseases (Supplementary Tables S3a–c). In addition, since the virtual populations in Simcyp™ are based on healthy subjects, a few PK studies in healthy volunteers were used for verification as well.
8. *What is the overall conclusion for gentamicin?* Our adjusted model-informed doses are likely to lead to more adequate *C*_max_ and *C*_trough_ levels. Remaining uncertainties, e.g., related to small changes in PK due to disease conditions and/or treatment [i.e., augmented CL in critically ill patients, decreased CL in congenital heart disease or hypothermia, increased Vd in extracorporeal membrane oxygenation (ECMO)] can be minimized by routine TDM, so that the optimized dose only contributes to better treatment of term neonates and infants.

## 4. Discussion

We used an already existing PBPK model of gentamicin to optimize neonatal and infant gentamicin doses. We showed that the current neonatal DPF dose for term neonates up to 1 month of age (4 mg/kg every 24 h) likely results in adequate *C*_max_ levels, but excessive, potentially toxic, *C*_trough_ levels. Additionally, for the youngest infants (4–6 weeks of age) the current infant DPF dose (7 mg/kg every 24 h) likely results in *C*_trough_ levels approximating the 1 mg/L limit. For older infants, a similar 7 mg/kg/24 h dose likely results in *C*_max_ levels at the low end of the therapeutic range. To achieve both adequate *C*_max_ as well as *C*_trough_ levels, model-informed dosing recommendations were developed (Table 1). These allow for a more gradual adjustment in dosing for term neonates and infants compared to the currently proposed dosing scheme in the DPF, which advocates a sudden increase in dose when a child reaches 1 month of age.

Before implementing dosing guidelines resulting from simulations, we advocate the use of a decision framework to clearly identify the assumptions while weighing the benefits and risks of the new dosing guideline (47). We identified the following assumptions: (1) *C*_max_/MIC is the best measure of efficacy for our population (32–36, 49, 50), 2) a *C*_max_ of 15–20 mg/L is required for infants and a *C*_max_ of 8–12 mg/L for neonates (33–41), (3) *C*_trough_ is the best predictor of toxicity (42–45), and (4) if a *C*_trough_ <1 mg/L is achieved, toxicity is prevented (7, 8, 18, 33–36, 42, 46, 51, 52). Combined with the answers on the questions asked within the decision framework, we evaluated the benefit-risk as positive.

Our findings are supported by a retrospective study evaluating 1,288 trough levels of 353 children aged 1 month to 17 years after a dose of 7 mg/kg every 24 h. They found high trough levels in only 2.2%, which is comparable to the 1.3% of our simulations (53). Another retrospective study in critically ill neonates showed that after a dose of 4 mg/kg every 24 h, 10 out of 34 (29%) trough levels measured were above 1 mg/L, which corresponds to the 5%–37% found in our simulations using the same dose (48). This study, and also another prospective study, reported lower proportions of therapeutic concentrations than we simulated using the study doses, but because of incomplete reporting of the conditions under which these values were determined, these data cannot be compared with our simulations (54). Both studies are unclear with regard to the exact sampling times used to determine *C*_max_. Although TDM standards aim to determine *C*_max_ 1 h after the start of infusion (i.e., 30 min after the end of infusion), these times may deviate in clinical practice. Sampling at later times will result in lower *C*_max_ values.

Another approach to study PK and determine dosing recommendations, is popPK. PopPK modeling is a top-down approach, where the PK data itself is used to fit the model. PBPK modeling is a bottom-up approach, starting with human physiology and drug characteristics (e.g., molecular weight, pKa, etc.). The virtual populations within the PBPK software are very well validated, which enables us to specifically investigate the effect of age-related physiological changes on gentamicin PK. Still, many attempts have been done to provide dosing recommendations using popPK. We found 18 popPK studies providing dosing recommendations for term neonates and/or infants (17, 18, 55–70). Only 4 of these studied the same population and the same therapeutic window as we did (55, 58, 61, 69). For neonates, only Valitalo et al. differentiated on postnatal age, recommending a 4.5 mg/kg dose every 48, 36, or 24 h for neonates ≤5 days, 6–10 days, or ≥11 days, respectively (69). The dose proposed by Bijleveld et al. applies only to neonates less than 7 days old and appears to be based largely on data from preterm neonates (58). For infants, large age ranges were included (i.e., up to 12 years of age) and the dose recommendations were based on MIC (55, 61). The model of Ghoneim et al., for example, aiming for a *C*_max_ of 20 mg/L, targeting an MIC of 2 mg/L, predicted that a dose of 6–7 mg/kg every 24 h according was required while the model of Alsultan et al. predicted that even a dose of 10 mg/kg would not be sufficient to achieve a *C*_max_ of at least 16 mg/L. According to Ghoneim et al. these differences could be explained by their additional age stratification as applied by Ghoneim et al. (61), which highlights the importance of our extensive PBPK modeling work in this population.

Our model-informed dose for neonates and infants until the age of 6 weeks is quite different from the current DPF dose. For older infants, only a minor dose increase is recommended, which is still important given the relatively large interindividual variability. Figure 2E shows that this variability covers half of the therapeutic window and increasing age is associated with lower mean plasma concentrations. The 5th percentile of the 7 mg/kg dose for infants aged ≥21 months and the 95th percentile of the 8 mg/kg dose for all infants falls outside the therapeutic window. Therefore, a dose of 7.5 mg/kg is most likely to achieve a therapeutic level.

Our study has some limitations. First of all, choosing another therapeutic target or window would logically affect the dose. Secondly, our dosing recommendations do not address variation in renal clearance or volume of distribution due to, for example, acute kidney injury, renal replacement therapy, or extracorporeal membrane oxygenation. PBPK modeling could be used for these purposes as well and should be explored in future research. A third limitation is the broad acceptance range. Besides the fact that this 2-fold range is the most commonly applied criterion, the large observed interindividual variability also justifies this range. Lastly, the model predictions for *C*_trough_ and *C*_max_ might appear biased for term neonates. For *C*_trough_, the higher predicted than observed concentrations reduce the risk of toxicity. For *C*_max_, the question arises as to whether the reported *C*_max_ is the “true *C*_max_”. A PBPK model can accurately predict *C*_max_, but sampling a few minutes too early or too late will always result in a lower observed *C*_max_.

In conclusion, our PBPK model was able to adequately capture pediatric PK of gentamicin. Our simulations indicate that until the age of 4 weeks postnatally, gentamicin should be administered at a dose of 4 mg/kg, but less frequently than currently recommended in the DPF. In order to achieve adequate *C*_max_ levels in infants from 4 weeks of age onwards, the current dose needs to be increased from 7 to 7.5 mg/kg, although the dose interval should be extended to 36 h for infants 4–6 weeks of age. Since TDM is advised for gentamicin, the effects of altered PK due to special co-morbidities, critical illness or ECMO, will still be noticed and the dose can be further adjusted accordingly. Evaluation according to the framework proposed by Hartman et al. showed a positive risk-benefit analysis for clinical implementation of this model-informed dose. With this study, we have shown that a pragmatic approach to establish model-informed dosages is feasible and that a framework to assess the readiness of model-informed dosing for clinical implementation is useful. In the future, we plan to apply this approach to many other drug to provide dosing recommendations for neonates, infants, children, and adolescents.

## Data Availability

The original contributions presented in the study are included in the article/**Supplementary Material**, further inquiries can be directed to the corresponding author.
