# Supplementary material for: Pragmatic physiologically-based pharmacokinetic modeling to support clinical implementation of optimized gentamicin dosing in term neonates and infants: proof-of-concept
Source: Front Pediatr. 2023 Nov 21;11:1288376. doi: 10.3389/fped.2023.1288376 (PMC10702772; doi:10.3389/fped.2023.1288376)
Supplement: Supplementary file 1 [file Datasheet1.docx]

Supplementary Material

Pragmatic physiologically-based pharmacokinetic modeling to support clinical implementation of optimized gentamicin dosing in term neonates and infants: proof-of-concept

Marika A de Hoop-Sommen^1^*, Joyce EM van der Heijden^1^, Jolien JM Freriksen^1^, Rick Greupink^1^, Saskia N de Wildt^1,2,3^

^1^Division of Pharmacology and Toxicology, Department of Pharmacy, Radboud University Medical Center, Nijmegen, The Netherlands, ^2^Department for Intensive Care, Radboud university medical center, Nijmegen, The Netherlands, ^3^Intensive Care and Pediatric Surgery, Erasmus MC, Rotterdam, The Netherlands

*** Correspondence:** Corresponding Author: [marika.dehoop-sommen@radboudumc.nl](mailto:marika.dehoop-sommen@radboudumc.nl)

**1. Supplementary Tables**

***Table S1.*** *Total daily doses recommended by several sources.*

|  |  | **Dose (mg/kg/day)** | | | | |
| --- | --- | --- | --- | --- | --- | --- |
| **Age** | **PNA** | **FDA(1)** | **EMA(2)** | **DPF(3)** | **BNF-C(4)** | **Lexi(5)** |
| <30 wk GA | 0-<7 d | 5* | *na* | 2.5▪ | *na* | 2.5▪ |
|  | 7-<15 d | 5* | *na* | 4 | *na* | 2.5▪ |
|  | ≥15 d | 5* | *na* | 4 | *na* | 3.33^◊^ |
| 30-<32 wk GA | 0-<7 d | 5* | *na* | 2.5▪ | *na* | 3.33^◊^ |
|  | 7-<15 d | 5* | *na* | 4 | *na* | 3.33^◊^ |
|  | ≥15 d | 5* | *na* | 4 | *na* | 3.33-5^◊◊^ |
| 32-<35 wk GA | 0-<7 d | 5* | *na* | 3.33^◊^ | *na* | 3.33^◊^ |
|  | 7-<15 d | 5* | *na* | 4 | *na* | 3.33^◊^ |
|  | ≥15 d | 5* | *na* | 4 | *na* | 3.33-5^◊◊^ |
| 35-<38 wk GA | 0-<7 d | 5* | *na* | 3.33^◊^ | *na* | 4 |
|  | ≥7 d | 5* | *na* | 4 | *na* | 5 |
| Term neonates | 0-<7 d | 5* | 4 | 4 | 3,33^◊^ | 4 |
|  | ≥7 d | 7.5° | 4 | 4 | 5 | 5 |
| Infants | 1-<24 mo | 7.5° | 4.5-7.5 | 7 | 7 | 4.5-7.5 |
| Children | 2-<12 y | 6-7.5° | 3-6 | 7 | 7 | 4.5-7.5 |
| Adolescents | 12-<18 y | 6-7.5° | 3-6 | 5-7 | 7 | 4.5-7.5 |
| Adults | ≥18 | 3-5° | 3-6 | *na* | *na* | *na* |

All dosages were converted to a mg/kg/24 h dose. In case the recommended dosing interval differs from every 24 h, this is indicated as follows: ▪ every 48 hours, ^◊^ every 36 hours, ^◊◊^ every 24-36 hours, * every 12 hours, ° every 8 hours. Abbreviations: BNF-C: British National Formulary for Children, d: day, DPF: Dutch Pediatric Formulary, EMA: European Medicines Agency, FDA: Food and Drug Administration, GA: Gestational Age, Lexi: Lexicomp Pediatric & Neonatal Dosage Handbook, mo: months, na: not available, PNA: Postnatal Age, wk: week, y: year

***Table S2.*** *Previously published PBPK model characteristics.*

|  | **Idkaidek et al. 2020(6)** | **Abduljalil et al. 2020(7)** | **Neeli et al. 2021(8)** | **Zazo et al. 2022(9)** |
| --- | --- | --- | --- | --- |
| Software | PK-Sim® | Simcyp® | Simcyp® | PhysPK platform |
| Distribution | Full | Full | Minimal | Minimal |
| V_ss_ (L/kg) | *na* | 0.31 | Adults: 0.17  Preterms: 0.5 | Term neonates: 0.46 ± 0.14  Preterms: 0.52 ± 0.16 |
| Population | Preterm | Preterm | Preterm | Preterm/term |
| Purpose study | Gentamicin levels  in saliva | Assess and verify  preterm model | PBPK-PD model | PBPK-PD model |
| Model-informed  dose? | No | No | PMA 30 – 34 weeks, PNA 8 – 28 days and PMA ≥35 weeks, PNA 0 – 7 days: 5 mg/kg q36h | Term neonates, <1 week PNA: 6 mg/kg q36h  Preterms, <1 week PNA: 6 mg/kg q48h |

Abbreviations: *na*: not available; PBPK-PD: physiologically-based pharmacokinetic – pharmaco-dynamic; PMA: post-menstrual age; PNA: postnatal age; q36h: every 36 hours; q48h: every 48 hours; V_ss_: volume of distribution at steady state.

***Table S3a.*** *Studies used for model verification; adults.*

| **Study** | **Design** | **Prop. of**  **females** | **Infusion**  **duration** | **N** | **Dose** | **Age range** | **Health status** | **Ref.** |
| --- | --- | --- | --- | --- | --- | --- | --- | --- |
| Boisson 2018 | SD | 0 | 30 min infusion | 12 | 8 mg/kg | 19 – 65 y | Critically ill | (10) |
| Choi 1999 | SD | 0.1 | 1 min infusion | 10^†^ | 80 mg | 22 – 50 y | Healthy | (11) |
| Demczar 1997 | SD | 0.82 | 1 h infusion | 11 | 2 mg/kg  7 mg/kg | 18 – 55 y | Healthy | (12) |
| Lewis 1999 | SD | NS | 10 min infusion | 9 | 4 mg/kg | 53.6 – 74.3 y | Cardiac surgery | (13) |
| Meunier 1987 | SD | 0.5 | Injection <1 min  15 min infusion | 10  10 | 80 mg  80 mg | 22 – 34 y | Healthy | (14) |
| Walker 1979 | SD | 0.3 | Bolus 3-5 min | 10^†^ | 120 mg | 22 – 32 y | Healthy | (15) |
| Liu 1999 | MD | 1 | 1 h infusion | 5 | 5 mg/kg, max 500 mg | 17 – 22 y | Postpartum endometritis | (16) |
| Triginer 1991 | MD | 0.4 | 30 min infusion | 10 | 3.5 mg/kg q24h | 32 – 76 y | Elective open heart surgery | (17) |

^†^A custom trial design was used. Abbreviations: h: hour, MD: multi dose, min: minutes, n: number, NS: not specified, q24h: every 24 hours, SD: single dose, y: year

***Table S3b.*** *Studies used for model verification; term neonates, infants, children and adolescents.*

| **Study** | **Design** | **Prop. of**  **females** | **Infusion**  **duration** | **N** | **Dose** | **Age** | **Health status (comorbidity)** | **Ref.** |
| --- | --- | --- | --- | --- | --- | --- | --- | --- |
| Bravo 1982 | SD | 0.71 | <5 min | 7^†^ | 3.5 mg/kg | 4-10 mo | Eutrophic* | (18) |
| Inparajah 2010 | SD | 0.36 | 30 min | 60  22  29 | 9.9 mg/kg^+^  7.9 mg/kg^+^  5.9 mg/kg^+^ | 1-<9 y  9-<12 y  ≥12 y | Febrile neutropenia (cancer) | (19) |
| MacDonald 1983 | SD | 0.5 | 30 min | 10 | 60 mg/m^2^ | 10-29 y | Cystic fibrosis | (20) |
| Shankar 1999 | SD | NS | 30 min | 8  10 | 6 mg/kg  6 mg/kg | 2-5 y  5-12.5 y | Infection or sepsis (cancer) | (21) |
| Agarwal 2002 | MD | NS | 30 min | 19  19 | 4 mg/kg q24h  2.5 mg/kg q12h | <24 h PNA | NICU patients | (22) |

^†^A custom trial design was used, *no clinical signs of infection, ^+^mean dose, ^median dose, **frequency of dosing is an assumption. Abbreviations: d: day, ECMO: extracorporeal membrane oxygenation, MD: multi dose, min: minutes, mo: months, n: number, NICU: neonatal intensive care unit, NS: not specified, PNA: postnatal age, qXh: every X hours, SD: single dose, y: year.

***Table S3b continued***

| **Study** | **Design** | **Prop. of**  **females** | **Infusion**  **duration** | **N** | **Dose** | **Age** | **Health status (comorbidity)** | **Ref.** |
| --- | --- | --- | --- | --- | --- | --- | --- | --- |
| Bass 1998 | MD | 0.44 | 4 min | 31  17 | 7.5 mg/kg q24h  2.5 mg/kg q8h | 0.5-18 y | Infection | (23) |
| Echeverria 1975 | MD | NS | 30 min | 60 | 2.54 mg/kg q8h  2.37 mg/kg q8h  1.97 mg/kg q8h  1.70 mg/kg q8h | 0.5-5 y  5-10 y  10-15 y  15-42 y | Neoplastic disease, cystic  fibrosis, infection | (24) |
| Evans 1978 | MD | NS | 20 min | 20 | 1.25 mg/kg or 37.5 mg/m^2^ q6h | 32-168 mo | Infection (cancer) | (25) |
| Evans 1980 | MD | 0.2 | 20 min | 50 | 37.5 – 60 mg/m^2^ q6h | 2.2-16 y | Infection (cancer) | (26) |
| Hansen 2003 | MD | NS | 30 min | 214 | 3 mg/kg q24h | <35 wk GA, 1-7 d PNA  ≥35 wk GA, 1-7 d PNA | NICU patients | (27) |
| Hoff 2009 | MD | NS | 30 min | 33 | 4 mg/kg q24h | 1 d PNA, >4000 g | Critically ill | (28) |
| Lares-Asseff 2016 | MD | 0.23 | 30 min | 26 | 2.5 mg/kg q8h | 3 mo – 5 y | Diarrhoea, pneumonia and septicaemia (malnutrition) | (29) |
| Low 2015 | MD | 0.33 | 30 min | 70 | 5 mg/kg q24h | ≤28 d PNA, mean 37.74 wk GA | Malaysian NICU patients | (30) |
| Moffett 2010 | MD | 0.35 | 30 min | 5  15  25  3 | 2.5 mg/kg q18h  2.5 mg/kg q8h  2.5 mg/kg q8h  1.7 mg/kg q8h | 15-26 d PNA  1.2-10.8 mo  1.1-11.2 y  13.1-16.8 y | Various  (congenital heart disease) | (31) |
| Paisley 1973 | MD | 0.09 | 20-30 min | 11^†^ | 0.95 mg/kg q6h^+,**^ | 34-41 wk GA, 1-23d PNA | Suspected sepsis | (32) |
| Rodriguez 2003 | MD | 0.4 | 30 min | 15 | 2.74 mg/kg q8h^+^  2.56 mg/kg q8h^ | 0.54-12 y | Infection (HIV) | (33) |
| Sawchuk 1976 | MD | NS | 60 min | 4 | 1.12-2.78 mg/kg q4h | 1.5-17 y | Sepsis (burns) | (34) |
| Shankar 1999 | MD | NS | 30 min | 36  37 | 7 mg/kg q24h | 2-5 y  5-12.5 y | Infection or sepsis (cancer) | (21) |
| Skopnik 1992 | MD | 0.7  0.5 | 30 min | 10  10 | 4 mg/kg q24h  2 mg/kg q12h | 1-3 d PNA | Infection | (35) |
| Southgate 1989 | MD | NS | 1 min | 10^†^ | 2.04-2.42 mg/kg q12h | 36-43 wk GA | NS (ECMO) | (36) |

^†^A custom trial design was used, *no clinical signs of infection, ^+^mean dose, ^median dose, **frequency of dosing is an assumption. Abbreviations: d: day, ECMO: extracorporeal membrane oxygenation, MD: multi dose, min: minutes, mo: months, n: number, NICU: neonatal intensive care unit, NS: not specified, PNA: postnatal age, qXh: every X hours, SD: single dose, y: year.

***Table S3c.*** *Studies used for model verification; preterm neonates.*

| **Study** | **Design** | **Prop. of**  **females** | **Infusion duration** | **n** | **Dose** | **Age** | **Health status** | **Ref.** |
| --- | --- | --- | --- | --- | --- | --- | --- | --- |
| Knight 2003 | MD | NS | 3-5 min | 31  142  94 | 2.5 mg/kg q24h  2.5 mg/kg q18h  2.5 mg/kg q12h | ≤28 wk GA, <4 d PNA  >28-<37 wk GA, <4 d PNA  ≥37 wk GA, <4 d PNA | Neonates requiring gentamicin | (37) |
| Hansen 2003 | MD | NS | 30 min | 214 | 3 mg/kg q24h | <35 wk GA, 1-7 d PNA  ≥35 wk GA, 1-7 d PNA | NICU patients | (27) |
| Husson 1984 | MD | NS | 60 min | 24 6 | 2.5 mg/kg q12h | ≥32 wk GA, <7 d PNA  ≥32 wk GA, >7 d PNA | Neonatal infection | (38) |

Abbreviations: d: day, GA: gestational age, MD: multi dose, min: minutes, n: number, NICU: neonatal intensive care unit, NS: not specified, PNA: postnatal age, qXh: every X hours, wk: week.

***Table S4.*** *Dosages prospectively simulated per age.*

| **PNA** | **Dose 1** | **Dose 2** | **Dose 3** | **Dose 4** | **Dose 5** |
| --- | --- | --- | --- | --- | --- |
| 1-28 days | 3 mg/kg | 3.5 mg/kg | 4 mg/kg | 4.5 mg/kg | 5 mg/kg |
| 4-6 weeks* | 7.5 mg/kg |  |  |  |  |
| 1-24 months | 6 mg/kg | 7 mg/kg | 7.5 mg/kg | 8 mg/kg | 9 mg/kg |

Neonatal dosages were simulated for the age 1, 7, 14, 21, and 28 days. Infantile dosages were simulated for every month of life until the age of 12 months. Thereafter, each dose is simulated for every additional 3 months (i.e., 15, 18, 21, and 24 months of age). *After data-analysis, no dose was found to result in both good C_trough_ and good C_max_ values for 1-month-old infants. Additional simulations were performed for infants 4, 5, and 6 weeks old. Abbreviation: PNA: postnatal age.

***Table S5.*** *Numbers of predicted-to-observed C_max_ and C_trough_ ratios within 2-fold (0.5-2) or bio-equivalence range (0.8-1.25).*

| **Age-group** | **C_max_** | | **C_trough_** | |
| --- | --- | --- | --- | --- |
|  | **0.5-2 range**  **(2-fold)** | **0.8-1.25 range**  **(bio-equivalence)** | **0.5-2 range**  **(2-fold)** | **0.8-1.25 range**  **(bio-equivalence)** |
| Adult | 7/7 (100%) | 5/7 (71%) | 3/5 (60%) | 2/5 (40%) |
| Adolescent | 9/9 (100%) | 8/9 (89%) | 4/6 (67%) | 2/6 (33%) |
| Child | 19/19 (100%) | 16/19 (84%) | 6/10 (60%) | 2/10 (20%) |
| Infant | 9/9 (100%) | 5/9 (56%) | 6/7 (86%) | 3/7 (43%) |
| Term neonate | 13/13 (100%) | 6/13 (46%) | 12/12 (100%) | 2/12 (17%) |
| Preterm neonate | 4/4 (100%) | 0/4 (0%) | 4/4 (100%) | 1/4 (25%) |
| All | 61/61 (100%) | 40/61 (66%) | 35/44 (80%) | 12/44 (27%) |
| Pediatrics (incl preterms) | 54/54 (100%) | 35/54 (65%) | 32/39 (82%) | 10/39 (26%) |
| Pediatrics (excl preterms) | 50/50 (100%) | 35/50 (70%) | 28/35 (80%) | 9/31 (29%) |

***Table S6.*** *Model-informed dose recommendations derived from simulated C_max_ and C_trough_ levels.*

|  | **Dose for desired C_max_ (mg/kg)** | **Interval for**  **desired C_trough_** | **Final dose recommendation** |
| --- | --- | --- | --- |
| 1 day | 3.5-4 | 48h | 4 mg/kg/48h |
| 7 days | 3.5-4 | 48h |  |
| 14 days | 3.5-4 | 36h (3.5 mg/kg)  48h (4 mg/kg) |  |
| 21 days | 3.5-4 | ≥36h | 4 mg/kg/36h |
| Infant 4-6 weeks | 7-7.5 | ≥36h | 7.5 mg/kg/36h |
| Infants 6 weeks-24 months | 7.5 | 24h | 7.5 mg/kg/24 |

**2. Supplementary Figures**


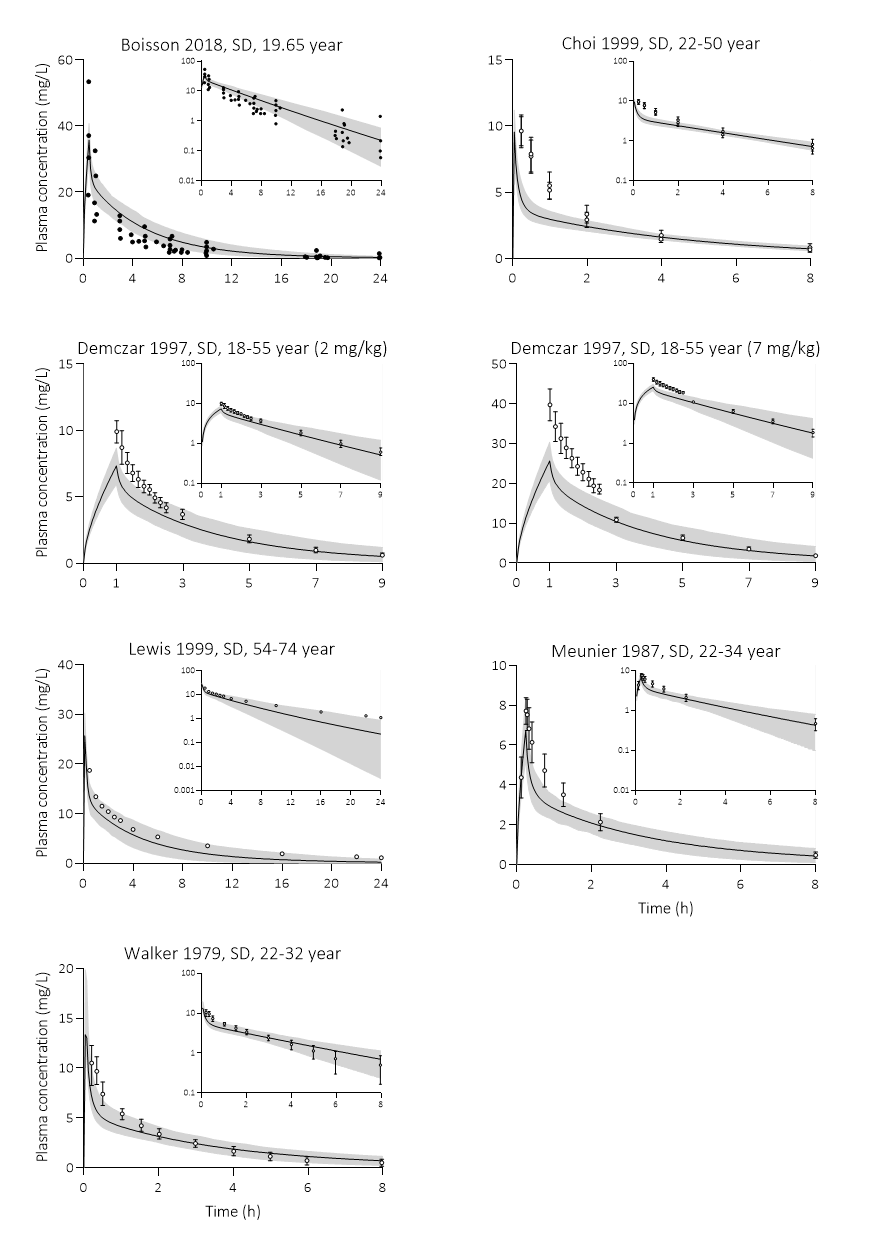


***Figure S1a.*** *Visual predictive checks of single doses (SD) of gentamicin in adults.* The solid line is the predicted mean of the simulated population and the shaded area represents the 5^th^ to 95^th^ percentile of the virtual population. Open circles are mean observed datapoints, closed circles are individual observed datapoints. Inserts show semi-log plots.


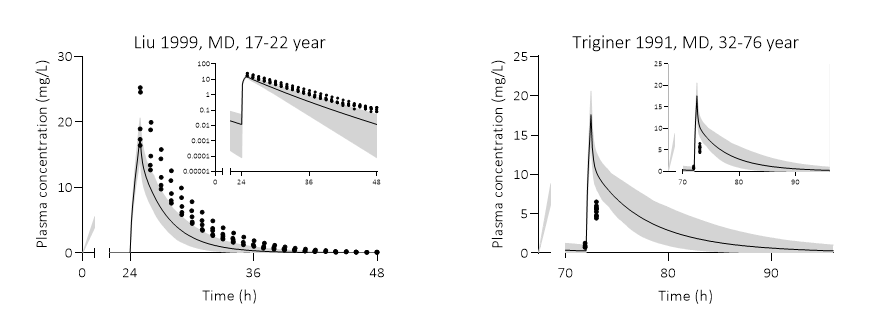


***Figure S1b.*** *Visual predictive checks of multiple doses (MD) of gentamicin in adults.* The solid line is the predicted mean of the simulated population and the shaded area represents the 5^th^ to 95^th^ percentile of the virtual population. Open circles are mean observed datapoints, closed circles are individual observed datapoints. Inserts show semi-log plots.


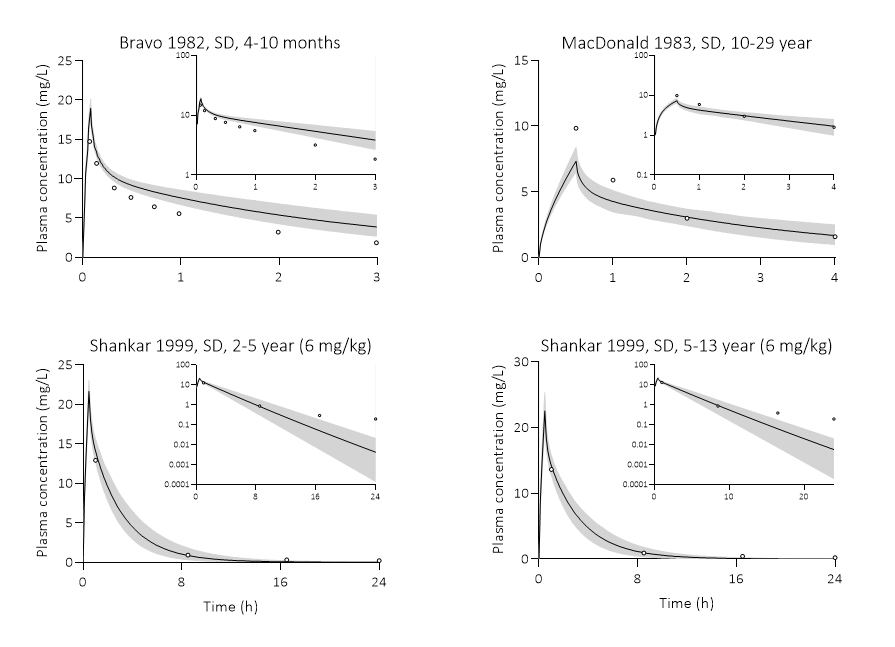


***Figure S2a.*** *Visual predictive checks of single doses (SD) of gentamicin in term neonates, infants, children, and adolescents.* The solid line is the predicted mean of the simulated population and the shaded area represents the 5^th^ to 95^th^ percentile of the virtual population. Open circles are mean observed datapoints, closed circles are individual observed datapoints. Inserts show semi-log plots.


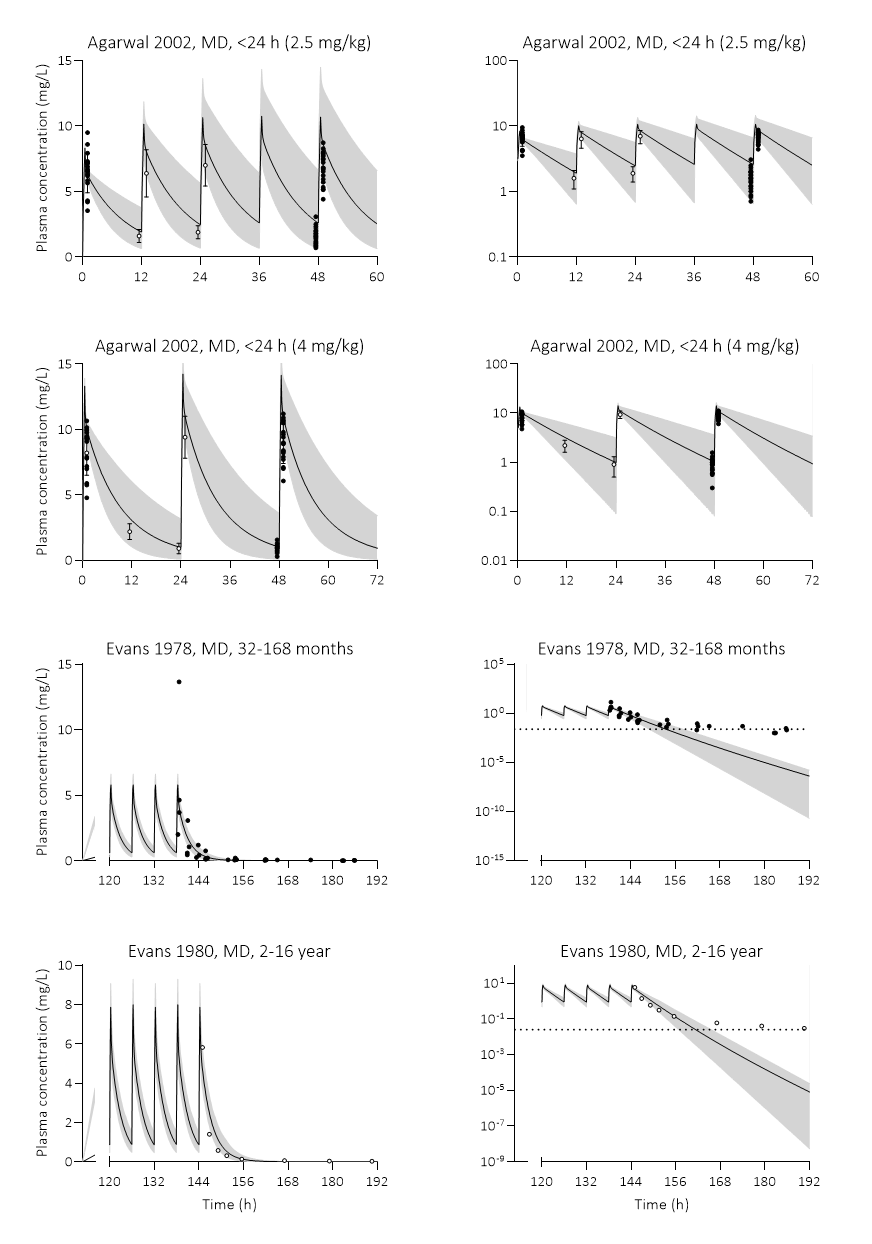


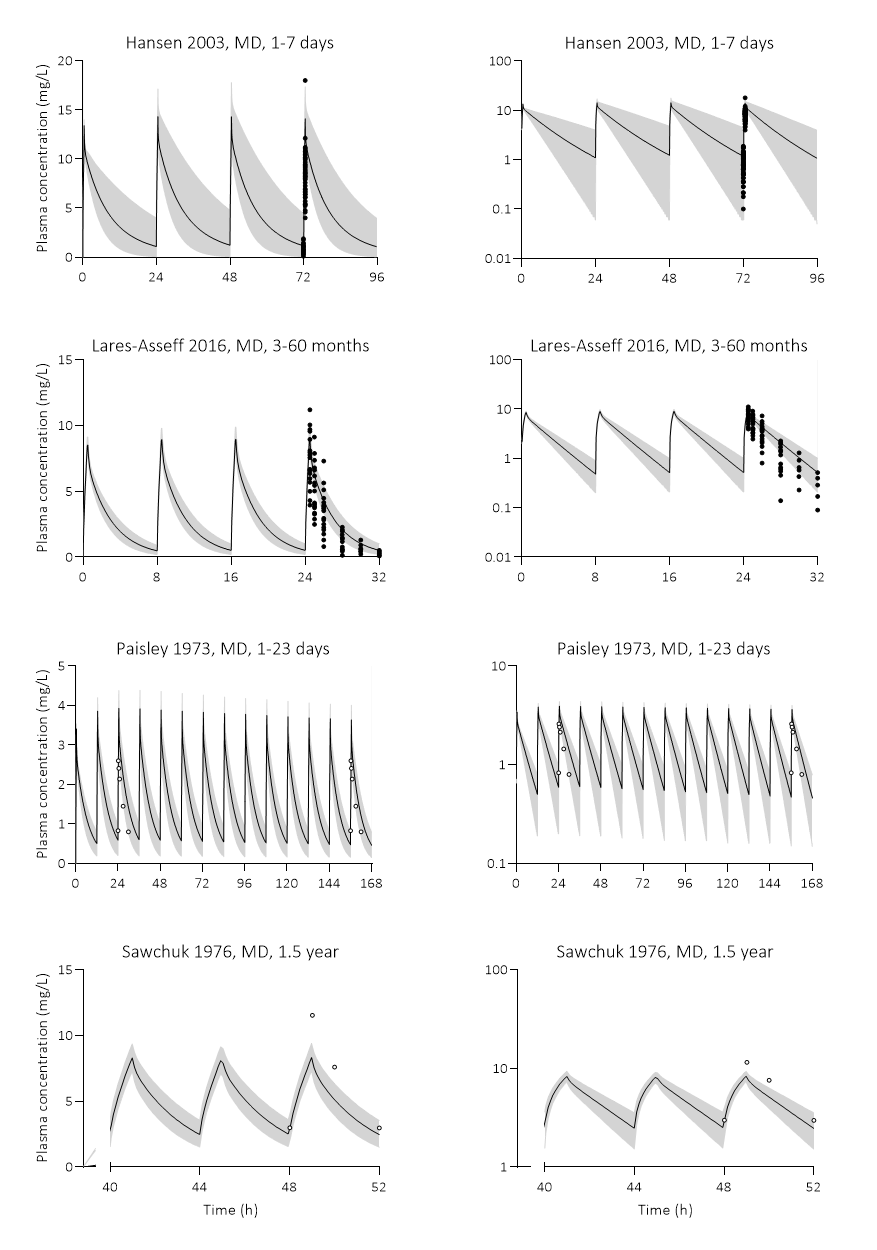


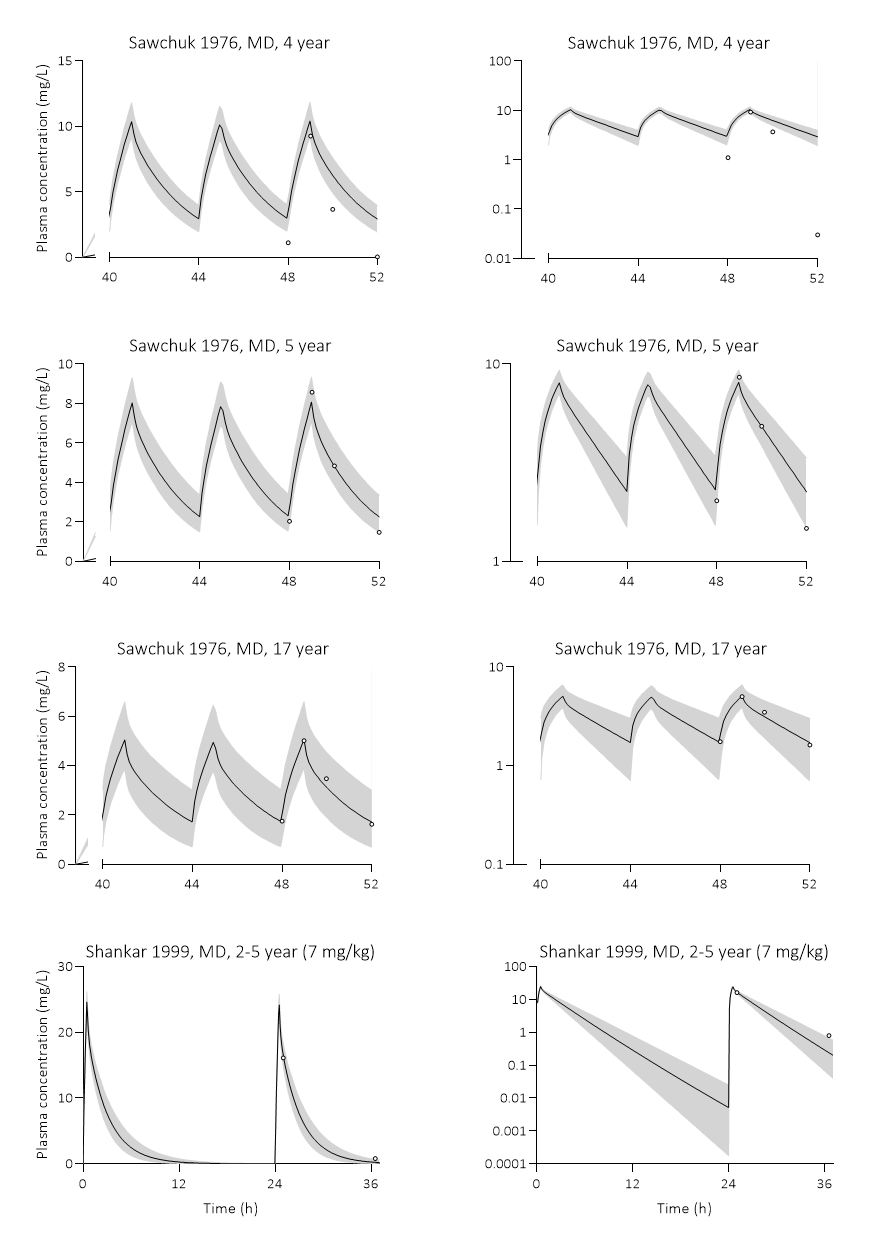


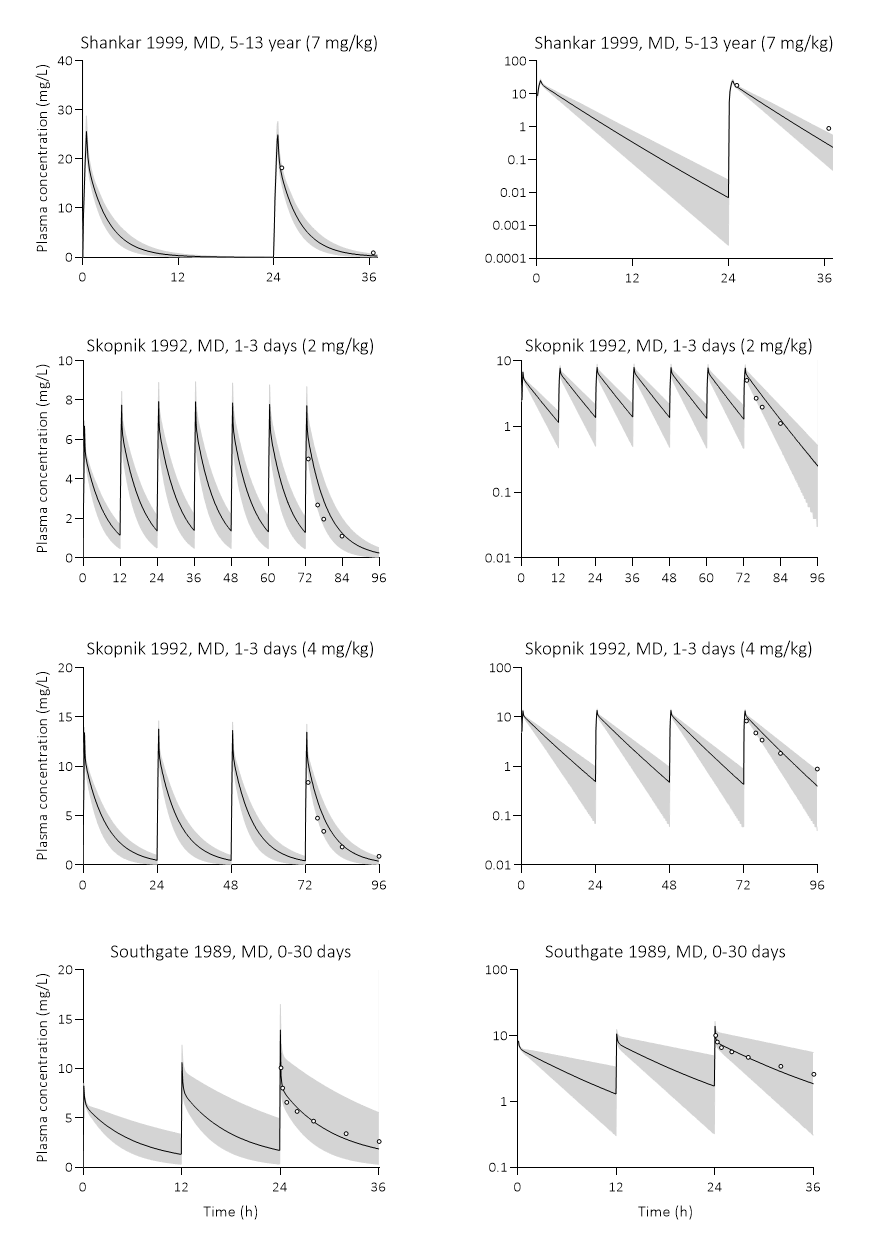


***Figure S2b.*** *Visual predictive checks of multiple doses (MD) of gentamicin in term neonates, infants, children, and adolescents.* The solid line is the predicted mean of the simulated population and the shaded area represents the 5^th^ to 95^th^ percentile of the virtual population. Open circles are mean observed datapoints, closed circles are individual observed datapoints. Dashed horizontal lines indicate the lower limit of quantification for the observed data points. On the left side linear plots are shown, on the right side the semi-log plots.


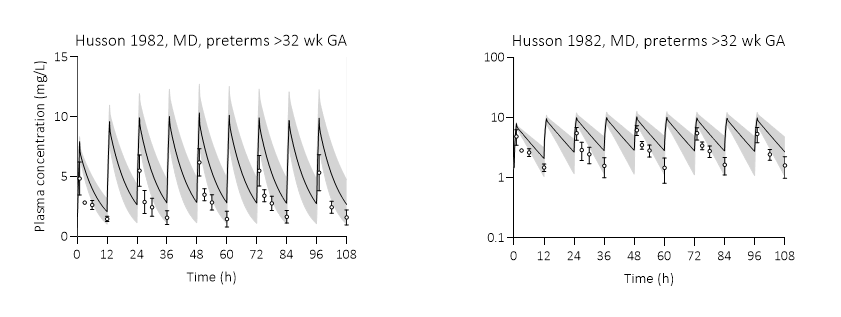


***Figure S3.*** *Visual predictive check of multiple doses (MD) of gentamicin in preterm neonates.* The solid line is the predicted mean of the simulated population and the shaded area represents the 5^th^ to 95^th^ percentile of the virtual population. Open circles are mean observed datapoints. On the left a linear plot, on the right a semi-log plot. Abbreviations: GA: gestational age, wk: weeks.

**3. References**

1. U.S. Food and Drug Administration. [Available from: <https://www.accessdata.fda.gov/scripts/cder/daf/>.

2. European Medicines Agency. [Available from: <https://www.ema.europa.eu/en/medicines>.

3. Dutch Pediatric Formulary [Available from: <https://www.kinderformularium.nl/>.

4. BMJ G. British National Formulary for Children. London: Pharmaceutical Press; 2020-2021.

5. Taketomo C, Hodding J. Lexicomp (R) Pediatric & Neonatal Dosage Handbook. 27th Edition ed: Lexi- Comp, Inc.; 2020.

6. Idkaidek N, Hamadi S, Bani-Domi R, Al-Adham I, Alsmadi M, Awaysheh F, et al. Saliva versus Plasma Therapeutic Drug Monitoring of Gentamicin in Jordanian Preterm Infants. Development of a Physiologically-Based Pharmacokinetic (PBPK) Model and Validation of Class II Drugs of Salivary Excretion Classification System. Drug research. 2020;70(10):455-62.

7. Abduljalil K, Pan X, Pansari A, Jamei M, Johnson TN. Preterm Physiologically Based Pharmacokinetic Model. Part II: Applications of the Model to Predict Drug Pharmacokinetics in the Preterm Population. Clinical pharmacokinetics. 2020;59(4):501-18.

8. Neeli H, Hanna N, Abduljalil K, Cusumano J, Taft DR. Application of Physiologically Based Pharmacokinetic-Pharmacodynamic Modeling in Preterm Neonates to Guide Gentamicin Dosing Decisions and Predict Antibacterial Effect. Journal of clinical pharmacology. 2021;61(10):1356-65.

9. Zazo H, Lagarejos E, Prado-Velasco M, Sánchez-Herrero S, Serna J, Rueda-Ferreiro A, et al. Physiologically-based pharmacokinetic modelling and dosing evaluation of gentamicin in neonates using PhysPK. Front Pharmacol. 2022;13:977372.

10. Boisson M, Mimoz O, Hadzic M, Marchand S, Adier C, Couet W, et al. Pharmacokinetics of intravenous and nebulized gentamicin in critically ill patients. J Antimicrob Chemother. 2018;73(10):2830-7.

11. Choi JS, Kim CK, Lee BJ. Administration-time differences in the pharmacokinetics of gentamicin intravenously delivered to human beings. Chronobiol Int. 1999;16(6):821-9.

12. Demczar DJ, Nafziger AN, Bertino JS, Jr. Pharmacokinetics of gentamicin at traditional versus high doses: implications for once-daily aminoglycoside dosing. Antimicrob Agents Chemother. 1997;41(5):1115-9.

13. Lewis DR, Longman RJ, Wisheart JD, Spencer RC, Brown NM. The pharmacokinetics of a single dose of gentamicin (4 mg/kg) as prophylaxis in cardiac surgery requiring cardiopulmonary bypass. Cardiovasc Surg. 1999;7(4):398-401.

14. Meunier F, Van der Auwera P, Schmitt H, de Maertelaer V, Klastersky J. Pharmacokinetics of gentamicin after i.v. infusion or iv bolus. J Antimicrob Chemother. 1987;19(2):225-31.

15. Walker JM, Wise R, Mitchard M. The pharmacokinetics of amikacin and gentamicin in volunteers: a comparison of individual differences. J Antimicrob Chemother. 1979;5(1):95-9.

16. Liu C, Abate B, Reyes M, Gonik B. Single daily dosing of gentamicin: pharmacokinetic comparison of two dosing methodologies for postpartum endometritis. Infect Dis Obstet Gynecol. 1999;7(3):133-7.

17. Triginer C, Izquierdo I, Fernández R, Torrent J, Benito S, Net A, et al. Changes in gentamicin pharmacokinetic profiles induced by mechanical ventilation. Eur J Clin Pharmacol. 1991;40(3):297-302.

18. Bravo ME, Arancibia A, Jarpa S, Carpentier PM, Jahn AN. Pharmacokinetics of gentamicin in malnourished infants. Eur J Clin Pharmacol. 1982;21(6):499-504.

19. Inparajah M, Wong C, Sibbald C, Boodhan S, Atenafu EG, Naqvi A, et al. Once-daily gentamicin dosing in children with febrile neutropenia resulting from antineoplastic therapy. Pharmacotherapy. 2010;30(1):43-51.

20. MacDonald NE, Anas NG, Peterson RG, Schwartz RH, Brooks JG, Powell KR. Renal clearance of gentamicin in cystic fibrosis. J Pediatr. 1983;103(6):985-90.

21. Shankar SM, Jew RK, Bickert BM, Cavalieri GE, Bell LM, Lange BJ. Pharmacokinetics of single daily dose gentamicin in children with cancer. J Pediatr Hematol Oncol. 1999;21(4):284-8.

22. Agarwal G, Rastogi A, Pyati S, Wilks A, Pildes RS. Comparison of once-daily versus twice-daily gentamicin dosing regimens in infants > or = 2500 g. J Perinatol. 2002;22(4):268-74.

23. Bass KD, Larkin SE, Paap C, Haase GM. Pharmacokinetics of once-daily gentamicin dosing in pediatric patients. J Pediatr Surg. 1998;33(7):1104-7.

24. Echeverria P, Siber GR, Paisley J, Smith AL, Smith DH, Jaffe N, et al. Age-dependent dose response to gentamicin. J Pediatr. 1975;87(5):805-8.

25. Evans WE, Feldman S, Barker LF, Ossi M, Chaudhary S. Use of gentamicin serum levels to individualize therapy in children. J Pediatr. 1978;93(1):133-7.

26. Evans WE, Taylor RH, Feldman S, Crom WR, Rivera G, Yee GC. A model for dosing gentamicin in children and adolescents that adjusts for tissue accumulation with continuous dosing. Clinical pharmacokinetics. 1980;5(3):295-306.

27. Hansen A, Forbes P, Arnold A, O'Rourke E. Once-daily gentamicin dosing for the preterm and term newborn: proposal for a simple regimen that achieves target levels. J Perinatol. 2003;23(8):635-9.

28. Hoff DS, Wilcox RA, Tollefson LM, Lipnik PG, Commers AR, Liu M. Pharmacokinetic outcomes of a simplified, weight-based, extended-interval gentamicin dosing protocol in critically ill neonates. Pharmacotherapy. 2009;29(11):1297-305.

29. Lares-Asseff I, Pérz-Guillé MG, Camacho Vieyra GA, Pérez AG, Peregrina NB, Lugo Goytia G. Population Pharmacokinetics of Gentamicin in Mexican Children With Severe Malnutrition. Pediatr Infect Dis J. 2016;35(8):872-8.

30. Low YS, Tan SL, Wan AS. Extended-interval gentamicin dosing in achieving therapeutic concentrations in malaysian neonates. J Pediatr Pharmacol Ther. 2015;20(2):119-27.

31. Moffett BS, Bork SJ, Mott AR. Gentamicin dosing for pediatric patients with congenital heart disease. Pediatr Cardiol. 2010;31(6):761-5.

32. Paisley JW, Smith AL, Smith DH. Gentamicin in newborn infants. Comparison of intramuscular and intravenous administration. Am J Dis Child. 1973;126(4):473-7.

33. Rodriguez JC, Schoenike S, Scott GB, Rossique-Gonzalez MT, Gomez-Marin O. An Evaluation of Gentamicin, Tobramycin, and Amikacin Pharmacokinetic/Pharmacodynamic Parameters in HIV-Infected Children. J Pediatr Pharmacol Ther. 2003;8(4):274-83.

34. Sawchuk RJ, Zaske DE. Pharmacokinetics of dosing regimens which utilize multiple intravenous infusions: gentamicin in burn patients. J Pharmacokinet Biopharm. 1976;4(2):183-95.

35. Skopnik H, Wallraf R, Nies B, Tröster K, Heimann G. Pharmacokinetics and antibacterial activity of daily gentamicin. Arch Dis Child. 1992;67(1 Spec No):57-61.

36. Southgate WM, DiPiro JT, Robertson AF. Pharmacokinetics of gentamicin in neonates on extracorporeal membrane oxygenation. Antimicrob Agents Chemother. 1989;33(6):817-9.

37. Knight JA, Davis EM, Manouilov K, Hoie EB. The effect of postnatal age on gentamicin pharmacokinetics in neonates. Pharmacotherapy. 2003;23(8):992-6.

38. Husson C, Chevalier JY, Jezequel M, Mathe JC, Costil J, Aymard P. Pharmacokinetic study of gentamicin in preterm and term neonates. Dev Pharmacol Ther. 1984;7 Suppl 1:125-9.
